# Structural and functional characterization of a cell cycle associated HDAC1/2 complex reveals the structural basis for complex assembly and nucleosome targeting

**DOI:** 10.1093/nar/gkv068

**Published:** 2015-02-04

**Authors:** Toshimasa Itoh, Louise Fairall, Frederick W. Muskett, Charles P. Milano, Peter J. Watson, Nadia Arnaudo, Almutasem Saleh, Christopher J. Millard, Mohammed El-Mezgueldi, Fabrizio Martino, John W.R. Schwabe

**Affiliations:** 1 Henry Wellcome Laboratories of Structural Biology, Department of Biochemistry, University of Leicester, Lancaster Road, Leicester LE1 9HN, UK; 2 MRC Laboratory of Molecular Biology, Francis Crick Avenue, Cambridge Biomedical Campus, Cambridge CB2 0QH, UK

## Abstract

Recent proteomic studies have identified a novel histone deacetylase complex that is upregulated during mitosis and is associated with cyclin A. This complex is conserved from nematodes to man and contains histone deacetylases 1 and 2, the MIDEAS corepressor protein and a protein called DNTTIP1 whose function was hitherto poorly understood. Here, we report the structures of two domains from DNTTIP1. The amino-terminal region forms a tight dimerization domain with a novel structural fold that interacts with and mediates assembly of the HDAC1:MIDEAS complex. The carboxy-terminal domain of DNTTIP1 has a structure related to the SKI/SNO/DAC domain, despite lacking obvious sequence homology. We show that this domain in DNTTIP1 mediates interaction with both DNA and nucleosomes. Thus, DNTTIP1 acts as a dimeric chromatin binding module in the HDAC1:MIDEAS corepressor complex.

## INTRODUCTION

The acetylation of histone tails plays a critical role in determining the accessibility of chromatin to transcriptional regulators and RNA polymerase complexes (1). Histone acetylation is a dynamic process that is controlled by the opposing action of lysine acetylase and deacetylase enzymes. The class I histone deacetylases (HDACs 1–3) are recruited to chromatin as large multi-component complexes that bring about transcriptional repression through chromatin compaction. These complexes typically contain, in addition to the HDAC catalytic domain, a scaffold protein responsible for recruitment of the complex to repressive transcription factors, as well as a number of proteins that mediate interaction with the chromatin substrate. The best understood of these complexes are the Sin3A, NuRD and CoREST complexes, which contain HDAC1 and/or HDAC2, and the SMRT/NCoR complex that contains HDAC3 (2–9). Recent reports have demonstrated that the catalytic activity of HDACs in the NuRD and SMRT/NCoR complexes is greatly enhanced as a result of inositol phosphates binding at the interface of the corepressor and HDAC catalytic domain (10,11). Characterizing HDAC complexes is an important goal toward understanding their role in the cell. Furthermore, drugs that target specific HDAC complexes are likely to have therapeutic advantages for the treatment of various diseases including cancer, HIV and Alzheimer's (12–14).

Recent proteomic studies have identified a novel HDAC1/2 complex in *Homo sapiens* and *Caenorhabditis elegans* called the MiDAC complex (15–17). In the human complex HDAC1/2 was found to be associated with MIDEAS (aka: C14orf43 and LOC91748) and/or the closely related proteins TRERF1 (aka: TReP-132, BCAR2, RAPA) and ZNF541 (aka: SHIP1). In the nematode complex, HDA-2 interacts with SAEG-1 (orthologues respectively of HDAC1/2 and MIDEAS). A third protein was identified in both the human and nematode complexes: DNTTIP1 (aka: TDIF1) and its orthologue SAEG-2 respectively (15–17). All of the components (HDAC1/2, MIDEAS and DNTTIP1) of the human MiDAC complex have been found to be specifically associated with cyclin A throughout the cell cycle (18). Furthermore, the MiDAC complex is upregulated in cells blocked in mitosis by treatment with nocodazole (15). However, the exact role of the complex in cell-cycle regulation remains to be elucidated.

DNTTIP1 was originally identified as a protein that is expressed in all tissue types and binds to the carboxy-terminus of TdT suggesting a role in regulating the recombination of immunoglobulin genes (19). There is also evidence that DNTTIP1 may serve as a regulator of transcription. Interestingly, DNTTIP1 is upregulated in the muscle of callipyge sheep leading to the increased activity of the promoter of MYH4, a myosin heavy chain associated with glycolytic muscle (20,21). DNTTIP1 has also been found to interact with TRERF1 (22). TRERF1 was originally described as a transcriptional coactivator protein (23,24). However, TRERF1 contains an ELM2-SANT domain that is found in co-repressor proteins such as MTA1, RCoR1, MIER and RERE, which act as scaffolds for several class I histone deacetylase complexes. We have recently reported the structure of a SANT domain from SMRT bound to HDAC3 (10) and an ELM2-SANT domain from MTA1 in complex with HDAC1 (11). These structures show how SANT domains bind to HDACs and establish that the ELM2 domain of MTA1 not only interacts with HDAC1 but mediates dimerization of the complex.

In this paper, we show that DNTTIP1 forms a stoichiometric complex with HDAC1 and the ELM2-SANT domain from MIDEAS. We report the structures of both the amino- and carboxy-terminal domains from DNTTIP1. The amino-terminal domain forms a novel dimerization domain, which we demonstrate interacts with the HDAC1/MIDEAS complex. The carboxy-terminal domain is structurally homologous to the SKI/SNO/DAC domain of the nuclear proto oncoprotein SKI and DACH1 proteins. Our experiments indicate that the SKI/SNO/DAC domain of DNTTIP1 binds directly to both naked DNA and to nucleosomes. Together these findings suggest that DNTTIP1 serves as a dimeric chromatin binding module in the MiDAC complex.

## MATERIALS AND METHODS

### Transient transfections

For expression in mammalian cells constructs of DNTTIP1, MIDEAS and HDAC1 were cloned into the pcDNA3 vector. The FLAG tagged constructs contained an N-terminal 10xHis-3xFLAG tag and a TEV protease cleavage site. HEK293F cells (Invitrogen) were co-transfected with mixtures of both tagged and untagged constructs using polyethylenimine (PEI) (Sigma). To transfect cells, 30 μg DNA total was diluted in 3 ml of PBS (Sigma) and vortexed briefly; 120 μl of 0.5 mg/ml PEI was added, and the suspension was vortexed briefly, incubated for 20 min at room temperature, then added to 30 ml cells (final density was 1 × 10^6^ cells/ml). Cells were harvested 48 h after transfection. For the interaction studies, the cells were lysed by sonication in buffer containing 50 mM Tris/Cl pH 7.5, 100 mM potassium acetate, 5% (v/v) glycerol, 0.3% (v/v) Triton X-100 and Complete EDTA-free protease inhibitor (Roche) (buffer A); the insoluble material was removed by centrifugation. The complex was then bound to FLAG resin (Sigma), washed three times with buffer A, three times with buffer B (50 mM Tris/Cl pH 7.5, 300 mM potassium acetate, 5% (v/v) glycerol) and three times with buffer C (50 mM Tris/Cl pH 7.5, 50 mM potassium acetate, 5% (v/v) glycerol, 0.5 mM tris(2-carboxyethyl)phosphine (TCEP)). The complex was eluted from the resin by overnight cleavage at 4°C with TEV protease in buffer C.

### Protein purification of full length DNTTIP1

Full length DNTTIP1(1–329) was cloned into pGEX2T (GE healthcare) and expressed in BL21 (DE3) (Novagen). Cells were grown at 37°C in 2xTY until OD_600nm_ = 0.6, induced with 0.5 mM Isopropyl β-d-1-thiogalactopyranoside(IPTG) followed by overnight growth at 15°C. The bacterial cells were lysed by sonication in a buffer containing 1x 10 mM Na2HPO4, 1.8 mM KH2PO4, 137 mM NaCl, 2.7 mM KCl (PBS), 1% Triton X-100, 1 mM Dithiothreitol (DTT) and Complete (Ethylenediaminetetraacetic acid) EDTA-free protease inhibitor (Roche). The soluble protein was bound to a glutathione sepharose column (GE healthcare), and washed with a buffer containing 1x PBS, 1% Triton X-100.

### Protease digestion

Full length GST-tagged DNTTIP1 (from 1 litre culture) on glutathione-sepharose (1 ml) was treated with 5 U thrombin (Sigma) with 2.5 mM CaCl_2_ at 4°C for 18 h or 0.2 μg trypsin (Sigma) at 21°C for 60 min. The supernatants were run on a sodium dodecyl sulfate-polyacrylamide gel electrophoresis (SDS-PAGE) gel followed by transfer to a PVDF membrane and N-terminal sequencing.

### Protein purification of the N-terminal dimerization domain (DD) and C-terminal DNA binding domain (DBD)

Based on the results of the protease digestion experiments and preliminary NMR experiments, human DNTTIP1 (56–147) (DD) and DNTTIP1 (197–316) (DBD) were cloned into pET30a (Novagen) with the addition of a TEV protease site and expressed in Rosetta-pLysS (Novagen). Cells were grown essentially as above or in a selenomethionine based medium in B834(DE3) or 2xTY for crystallization or in a M9-based medium (supplemented with ^15^NH_4_Cl or ^15^ NH_4_Cl and ^13^C-glucose (Cambridge isotopes)) for NMR. Cells were lysed by sonication in buffer A containing 20 mM Tris/Cl pH 7.0, 1 mM 4-(2-Aminoethyl) benzenesulfonyl fluoride hydrochloride (AEBSF), 2 mM TCEP and Complete EDTA-free protease inhibitor (Roche). After centrifugation the supernatant was applied to a Ni-NTA agarose column (QIAGEN), and the His-tagged protein was eluted with buffer A containing 250 mM imidazole. The His-tag was removed by overnight digestion with TEV protease at 22°C.

The N-terminal domain was further purified on a Resource Q column (GE Healthcare) using a 0–0.5 M NaCl gradient in buffer A followed by gel filtration on a Superdex S75 in 20 mM Tris/Cl pH 8.0, 1 mM 4-(2-Aminoethyl) benzenesulfonyl fluoride hydrochloride (AEBSF), 2 mM tris(2-carboxyethyl)phosphine (TCEP) and Complete EDTA-free protease inhibitor (Roche). The protein was concentrated to 40 mg/ml for crystallization experiments.

The C-terminal domain was futher purified on a Resource S column (GE Healthcare) using a 0–0.5 M NaCl gradient in buffer A followed by gel filtration on a Superdex S75 column in 40 mM potassium/sodium phosphate pH 6.0, 1 mM DTT and 1 mM NaN_3_. The protein was finally concentrated to 0.3 mM in 40 mM K/Na phosphate pH 6.0, 1 mM DTT, 1 mM NaN_3_ with 10% D_2_O or 100% D_2_O for the NMR experiments.

### Crystallization of N-terminal dimerization domain

Crystallization was carried out using a vapor-diffusion method at 22°C. Crystals of DNTTIP1 (56–147) were grown in hanging drops of a mixture of 1.0 μl of protein solution and 1.0 μl of reservoir solution containing 100 mM sodium acetate pH 4.6, 14% propan-2-ol. SeMet substituted protein was crystallized using 100 mM sodium acetate pH 4.9, 14% ethoxyethanol. Both crystal types were *P*2_1_2_1_2_1_ with cell dimensions *a* = 54.91 Å, *b* = 103.05 Å, *c* = 108.93 Å.

### Crystallographic data collection and processing

The native and multiwavelength anomalous dispersion (MAD) datasets were both collected at ID 23.1 ESRF, Grenoble. The native crystals were cryoprotected in 100 mM sodium acetate pH 4.6, 20 mM Tris/Cl pH 8.0, 2 mM DTT, 14% propan-2-ol and 10% glycerol. The SeMet crystals were cryoprotected with 50% paratone and 50% parafin equilibrated in 100 mM sodium acetate pH 4.9, 20 mM Tris/Cl pH 8.0, 2 mM DTT and 14% ethoxyethanol. Both crystal types were frozen in a N_2_ stream at 100 K. Data processing was performed in MOSFLM (25). Subsequent crystallographic computations were performed using CCP4 suite of programs, Coot and REFMAC were used for model building and refinement (26,27). The data processing and refinement statistics are shown in Supplementary Table S1.

### NMR chemical shift assignment

All NMR data were acquired at 25°C on either 600 or 800 MHz Bruker DRX/AvanceII systems with cryogenically cooled probeheads. The 2D and 3D spectra recorded to obtain sequence specific assignments were: ^15^N/^1^H HSQC; NOESY-HSQC with a mixing time of 100ms; ^13^C/^1^H HSQC; CCH-TOCSY and HCCH-TOCSY with a mixing time of 13 ms, ^13^C/^1^H NOESY-HSQC with an NOE mixing time of 100 ms, HNCACB, CBCA(CO)NH, HN(CO)CA, HNHA, HBHA(CO)NH, HNCA and HNCO (reviewed in (28)). All NMR data were processed using Topspin (Bruker Biospin Ltd) and analyzed using the Sparky package (T.D. Goddard and D.G. Kneller, Sparky 3, University of California, San Francisco, USA).

### Structure calculations

The family of converged structures was determined in a two stage process using the program CYANA (29), as described previously (30). Hydrogen bond constraints, involving 13 residues with slowly exchanging backbone amide signals and where the hydrogen bond acceptor was unambiguous in preliminary structures, were added to the final round of calculations. Backbone torsion angle constraints derived from the protein backbone dihedral angle prediction program TALOS+ (31) were included in both stages of the calculation. The 65 structures that contained no distance or van der Waals violation higher than 0.5 Å and no dihedral angle violations higher than 5° were selected for analysis. The NMR constraints and structural statistics for DNTTIP1 (197–316) are summarized in Supplementary Table S2. Analysis of the final family of structures obtained was carried out using PROCHECK-NMR (32) and MOLMOL (33).

### Electrophoretic mobility shift assays

The electrophoretic mobility shift assay probe mixture of double stranded DNA was prepared by annealing complementary oligonucleotides to the following sequences:

5′CATAGTCAGGTCAGGTCAGATCAAC3′
5′CATAGTCAGGTCAAGGTCAGATCAAC3′
5′CATAGTCAGGTCATAGGTCAGATCAAC3′
5′CATAGTCAGGTCAATAGGTCAGATCAAC3′
5′CATAGTCAGGTCATATAGGTCAGATCAAC3′

The double stranded DNA mixture of 1 and 5 μM was incubated with a one to ten-fold excess of DNTTIP1 (DBD197-316) in binding buffer (20 mM HEPES pH 7.0, 75 mM KCl, 0.1% Triton X-100, 7.5% glycerol, 2 mM DTT) for 20 min on ice. Samples were analyzed on a 10 cm x 10 cm, 5% acrylamide gel buffered in 0.5x TB (45 mM Tris, 45 mM boric acid) run at 60 V for 60 min at 4°C. The gel was stained with ethidium bromide and visualized using UV.

### NMR titration of DNA binding

The titration of DNTTIP1 (100 μM) with the mixed DNA (100–1000 μM) (as prepared above) was monitored by performing a ^15^N/^1^H HSQC spectrum at each titer point.

### Circular dichroism experiments

For the circular dichroism experiments, the concentration of DNTTIP1 dimerization domain was 50 μM and DNA binding domain was 20 μM in 10 mM K/Na phosphate buffer, 1 mM DTT. Circular dichroism spectra were measured from 200 to 250 nm at various temperatures. For the melting curves, circular dichroism was monitored at 222 nm, with data points collected as the sample temperature was increased from 5 to 95°C (1°C/min) using a Jasco J-715 spectropolarimeter with a Jasco PTC-348WI temperature controller. An approximate melting temperature was obtained by fitting the data to a single-site sigmoidal dose response curve (GraphPad Prism).

DNTTIP1 DBD (20 μM) was incubated with 60 μM of the DNA mixture (as prepared above) on ice for 20 min in the buffer (10 mM K/Na phosphate buffer, 1 mM DTT). Circular dichroism spectra and melting curves were measured with and without the DNA.

### Nucleosome binding assays

Nuclei were purified from 1 ml of turkey blood by lysis and washing in phosphate-buffered saline (PBS) pH 7.2, 0.2 M NaCl, 0.5% Triton X-100 and Complete EDTA-free protease inhibitor (Roche). To digest with micrococcal nuclease the nuclei were resuspended in 10 mM Tris/Cl pH 8.0, 0.3 M, NaCl, 1 mM CaCl_2_ with 100 U of micrococcal nuclease. This was incubated at 37°C for 30 min. The enzyme was stopped with the addition of EDTA to 2 mM. Short chromatin was in the supernatant after a brief spin.

GST-tagged proteins were expressed and purified as above. GST tagged protein from 100 ml of *Escherichia coli* bound to 100 μl Glutathione sepharose was incubated with 100 μl of chromatin with 1 ml 20 mM Tris/Cl pH 8.0, 100 mM NaCl, 0.5 mM DTT, 0.1% Triton X-100 for 20 min. This was then washed three times with the same buffer. Bound proteins were eluted in SDS-PAGE gel loading buffer and visualized by SDS-PAGE.

### Large scale purification of the HDAC1/MIDEAS/DNTTIP1 complex

The HDAC1/MIDEAS/DNTTIP1 complexes were purified from 1.2 l of HEK293F cells expressed as described above but with 300 ml of cells in 2 l roller bottles. The cells were lysed by sonication in buffer containing 50 mM Tris/Cl pH 7.5, 100 mM potassium acetate, 10% (v/v) glycerol, 0.5% (v/v) Triton X-100, and Complete EDTA-free protease inhibitor (Roche) (buffer A); the insoluble material was removed by centrifugation. The complex was then bound to FLAG resin (Sigma), washed twice with buffer A, three times with buffer B (50 mM Tris/Cl pH 7.5, 50 mM potassium acetate, 5% (v/v) glycerol, 0.5 mM TCEP), incubated with 0.5 mg RNaseA for 1 h at 4°C and then washed five times with buffer B. The complex was eluted from the resin by overnight cleavage at 4°C with TEV protease in buffer B. The complex was further purified by gel filtration on a Superdex-200 column (GE Healthcare).

### Chromatin reconstitutions

Chromatin was reconstituted optimizing a previously published method (34). Briefly, the 601 DNA, the competitor DNA and the histone octamer were mixed in 2 M NaCl, 10 mM triethanolamine pH 7.4, 1 mM EDTA and dialyzed at 4°C as follows: 3 h 1.2 M NaCl, 3 h 1 M NaCl, 3 h 0.8 M NaCl, O/N 0.6 M NaCl, O/D 0.3 M NaCl, O/N 50 mM NaCl. Trace amounts of cy5-labeled 601 DNA were added to the reaction to better monitor the formation of NCPs. The amount of histone octamer required to reach saturation was empirically determined by titrating a constant amount of 601 nucleosome positioning DNA sequence with increasing concentrations of recombinant *Xenopus laevis* histone octamer. Competitor DNA was added to the reconstitution at a ratio 1:2 to prevent super-saturation of the 601 DNA. Competitor increases the chromatin solubility by sequestering the histones excess while promoting full occupancy of the 601 DNA. After reconstitution, NCPs were loaded on a native agarose gel run in a 0.2x TB buffer (18 mM Tris, 18 mM boric acid). The gel was analyzed for cy5 fluorescence emission and ethidium bromide stained to monitor, respectively, the loading of the octamer on the cy5-labeled 601 DNA and on the competitor DNA. Only saturated NCPs were used in biochemical assays. When making mono-nucleosomes the pUC competitor is completely saturated by nucleosomes as described in (35). Arnaudo *et al*. (35) purified mono-nucleosomes by selectively precipitating the pUC poly-nucleosomes with MgCl_2_ and PEG (35).

Interactions of the reconstituted chromatin with full-length HDAC1, full-length DNTTIP1 and MIDEAS (650–887) were analyzed on a 10 cm x 10 cm, 0.7% agarose gel buffered in 0.5x TB (45 mM Tris, 45 mM boric acid) run at 30 mA for 30 min. The gels were stained with ethidium bromide and visualized using UV.

### Chromatin purification from HEK293F cells

Chromatin was prepared from HEK293F nuclei essentially as described in (36). Nuclei at 50 *A*_260_/ml were digested with 50 U /ml micrococcal nuclease for 20 min at 37°C. The nuclei were lysed in 1 mM Tris/Cl pH 7.5, 0.2 mM EDTA. Soluble chromatin was fractionated in 5–50% sucrose gradients in 20 mM Tris/Cl pH 7.5, 60 mM NaCl, 0.2 mM EDTA.

### HDAC activity assays

HDAC activity assays carried out as described in (10). To perform the titration, 62 nM protein was incubated with varying concentrations of inositol-1,4,5,6-tetraphosphate and inositol-1,4,5-triphosphate for 30 min at 37°C then the HDAC activity was measured using the HDAC Assay Kit (Active Motif) and read using a Victor X5 plate reader (PerkinElmer). Experiments were performed in triplicate and data were analyzed using GraphPad Prism (version 4.0, GraphPad Software, Inc.).

### Mass spectrometry

FLAG tagged DNTTIP1 was expressed in HEK293F and purified as above except that 50 mM Tris/Cl pH 8 and 150 mM NaCl were used. Proteomics was carried out by the University of Leicester Proteomics Facility (PNACL, University of Leicester) essentially as described previously (37). The TEV eluted protein was run just into the top of the separating SDS-PAGE gel and the mixture of proteins cut out of the gel as a single band. The gel slice was digested with trypsin and subjected to LC-MS/MS mass spectrometry using an RSLCnano HPLC system (Dionex, UK) and an LTQ-Orbitrap Velos mass spectrometer (Thermo Scientific). The data were analyzed using Mascot (Matrix Science Ltd) and Scaffold (Proteome Software).

## RESULTS

### A stoichiometric complex formed by DNTTIP1, MIDEAS and HDAC1

**Figure 1.**
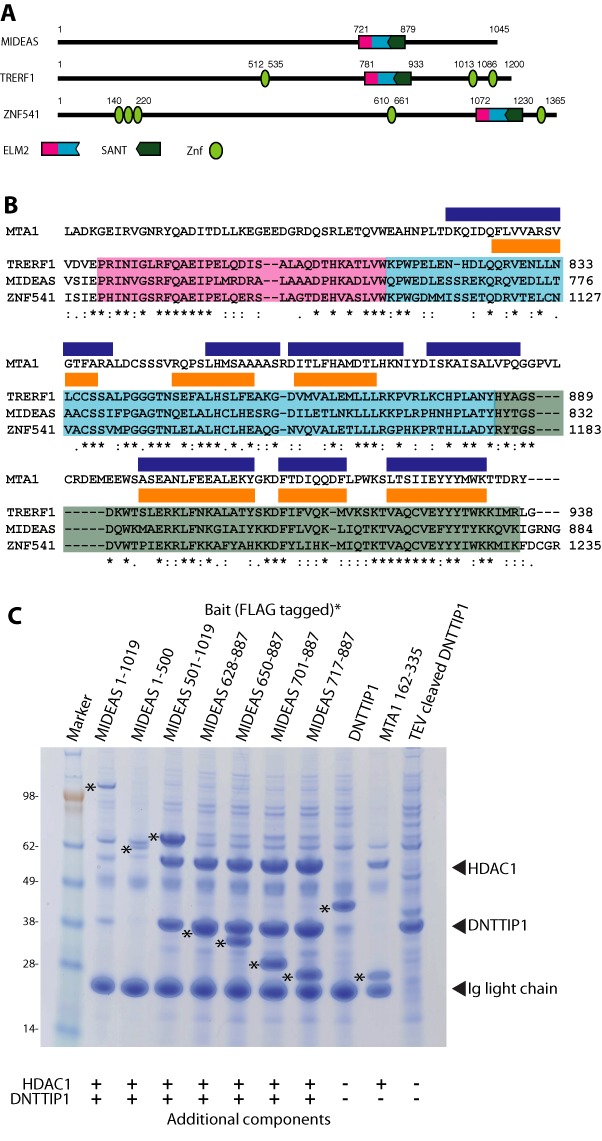
Interaction of DNTTIP1 with histone deacetylase complexes. (**A**) Domain structures of MIDEAS, TRERF1 and ZNF541; three co-repressor proteins associated with DNTTIP1. (**B**) Clustal alignment of ELM2 (magenta and cyan) and SANT (green) domains of TRERF1, MIDEAS and ZNF541 compared with the ELM2-SANT domain of MTA1. The blue rectangles are the α-helices in MTA1 and the orange rectangles are the predicted α-helices in MIDEAS. (**C**) Small scale co-transfection of HEK293F cells and purification of various FLAG tagged constructs of MIDEAS (marked with an asterisk) with full-length DNTTIP1 and full-length HDAC1. FLAG-tagged DNTTIP1 and MTA1 (also marked with an asterisk) with untagged HDAC1 and TEV cleaved FLAG-tagged DNTTIP1 are shown as controls.

It has recently been shown using pull-down experiments from human K562 and HEK293F cells that DNTTIP1 interacts with three human ELM2-SANT containing proteins, TRERF1, MIDEAS and ZNF541 (Figure 1A) (15–17). Our own mass spectrometry experiments with transiently expressed FLAG tagged DNTTIP1 also show that DNTTIP1 pulls down MIDEAS and HDAC1 (Supplementary Table S3). Comparison of the three DNTTIP1 interacting proteins shows that the most conserved region is the ELM2-SANT domain (Figure 1B). Based on our recent structure of the ELM2-SANT domain from MTA1 bound to HDAC1 (11), we would expect TRERF1, MIDEAS and ZNF541 to also interact directly with HDAC1 and/or HDAC2. Indeed, the proteomics studies clearly indicated association with both HDAC1 and HDAC2 (15–17).

To determine whether it is the ELM2-SANT domain itself that mediates interaction with DNTTIP1, we co-expressed various FLAG tagged constructs of MIDEAS with both DNTTIP1 and HDAC1 in HEK293 cells. We found that the ELM2-SANT domain of MIDEAS is sufficient to form a tight stoichiometric ternary complex with DNTTIP1 and HDAC1 (Figure 1C). In addition, we found that all three components are required for the purification of a stable complex (Supplementary Figure S1).

### DNTTIP1 contains two independently folded domains

**Figure 2.**
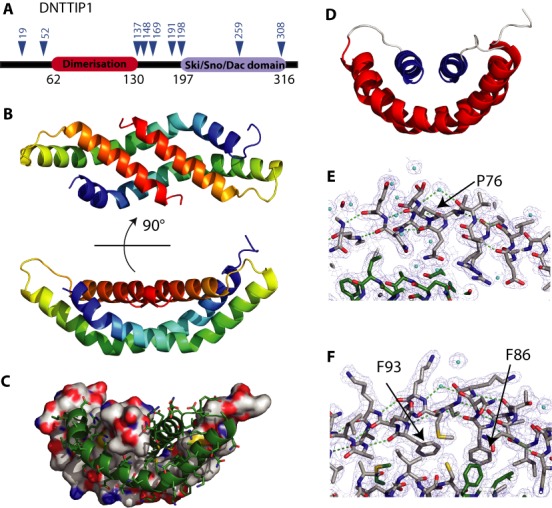
The crystal structure of the dimerization domain of DNTTIP1. (**A**) Domain structure of DNTTIP1. The protease digestion sites are shown as blue triangles. (**B**) Cartoon representation of the dimer of DNTTIP1 (62–130) in two different orientations 90° apart. The proteins are colored from the N- to C- terminus from blue to red. (**C**) The extensive dimeric interface between the two monomers with one molecule shown as a surface representation bound to a cartoon and sticks representation of the other molecule. (**D**) An alternative orientation of the dimerization domain. (**E** and **F**) Water molecules (shown in cyan) stabilizing the two kinks in the long helix 1. The hydrogen bonds from the peptide backbone of the α-helix are shown in green. The water molecules are shown in cyan.

Full-length GST-tagged DNTTIP1 could be readily expressed in *E. coli*. However, the protein was found to be sensitive to cleavage by endogenous proteases during purification and by thrombin protease during removal of the GST tag. Amino-terminal sequencing of the thrombin derived fragments identified amino-termini of Pro137, Gly149, Arg169 and Ser191 (shown in Figure 2A). To characterize the potential domains further we digested the full-length GST-tagged DNTTIP1 using trypsin. Amino-terminal sequencing of the resulting fragments identified amino-termini of Arg53, Ser54 and Glu198 and Ala260. Taken together, these data suggest that there are two folded domains within DNTTIP1. We found that constructs of DNTTIP1 (56–147) and DNTTIP1 (197–316) were soluble, express to a high level and could be readily purified.

### A novel amino-terminal dimerization domain

Human DNTTIP1 (56–147) crystallized with six molecules within the asymmetric unit. The structure was solved at 2.1 Å resolution using MAD phasing with seleno-methionine substituted protein (Supplementary Table S1). It was immediately clear in the electron density that the protein was arranged in the asymmetric unit as three dimers. Each monomer consists of a long amino-terminal helix divided into three by two distinct kinks. Carboxy-terminal to this long helix, the peptide chain folds back on itself leading into a second, shorter helix arranged at ∼60° to the central portion of the longer helix. The dimer is arranged such that the longer helices in the two monomers lie anti-parallel to each other and form a saddle into which the two shorter carboxy-terminal helices are packed together, also in an anti-parallel orientation (Figure 2B). This arrangement results in a large buried surface between the two monomers of 4083 Å^2^. Remarkably, this represents 69% of the whole surface of the monomer (Figure 2C). The Dali protein structure comparison server (38) identifies no structures that are significantly similar to the DNTTIP1 dimerization domain suggesting that this is a novel fold (Figure 2D).

In each of the kinks in the longer helix, a water molecule is positioned such that it makes hydrogen bonds to the peptide backbone that would normally be satisfied by the helical secondary structure. This has been seen in other structures (reviewed in (39)) and clearly stabilizes the backbone conformation. At one kink, a proline residue (Pro76) naturally distorts the helix (Figure 2E). The backbone trajectory at the other kink, seems to be caused through the need to bury two phenylalanines (Phe86 and Phe93) on either side of the kink (Figure 2F). The core of the interface between the monomers consists entirely of non-polar amino acid side chains. In addition to the non-polar core, there are many polar and electrostatic interactions on the surface of the monomers that bridge the dimer interface.

The structure and extensive buried surface at the dimer interface, suggests that the DNTTIP1 dimerization domain is a particularly tight interaction. To assess the stability we performed Circular Dichroism melting experiments which indicate that the domain unfolds at 67°C and furthermore that the protein can be melted and renatured multiple times without any material becoming irreversibly denatured (Supplementary Figure S2). The protein will renature fully on both slow-cooling and snap-cooling from 95 to 5°C. We conclude therefore, that this is a particularly stable and readily refolded dimerization domain that may have biotechnological applications.

### A carboxy-terminal SKI/SNO/DAC domain

**Figure 3.**
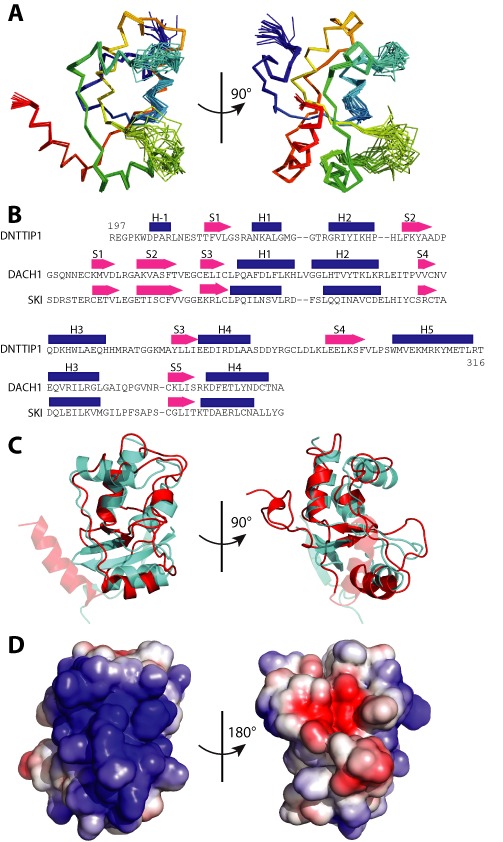
The NMR structure of the DNA binding domain of DNTTIP1. (**A**) Two views of DNTTIP1 (197–316) oriented at 90° to each other of a superposition of the Cα atoms for the 20 lowest energy structures of the 65 that converged are shown colored N to C terminus from blue to red. (**B**) Structural alignment of the primary sequence of the SKI/SNO/DAC domains of human DNTTIP1, SKI and DACH1. Strands are shown as pink arrows and helices as blue rectangles. (**C**) Superposition of DNTTIP1 (197–316) (red) with the SKI/SNO/DAC domain of the Ski oncogene (cyan). The elements of DNTTIP1 (H-1), and of SKI (S1), that do not superimpose are slightly transparent. (**D**) Two views oriented at 180° to each other of the electrostatic potential of the surface of DNTTIP1 (197–316) calculated in APBS (45) (blue is positive and red is negative).

The structure of the carboxy-terminal domain of DNTTIP1 (197–316) was determined by NMR spectroscopy. The RMSD for the backbone of the amino acids 199–315 was 0.86 Å^2^. DNTTIP1 (197–316) forms a compact α/β structure. The core of the structure is a well-converged four stranded twisted anti-parallel β-sheet with the strands interspersed with α-helices (Figure 3A).

The Dali protein structure comparison server (38) revealed significant matches to SKI-like protein (3eq5), human DACH1 (1lr8) (40) and the SKI oncogene (1sbx) (41) with *Z*-scores of 4.5, 4.2 and 4.1, respectively. The lowest RMSD (2.6 Å^2^) is with the SKI-oncogene. A structural alignment using LSQman (Uppsala Software factory) shows that the core of the carboxy-terminal domain of DNTTIP1 superimposes with the SKI/SNO/DAC family (PFam PF02437) (Figure 3B and C). DNTTIP1, however, lacks the CLPQ motif that has been suggested to be characteristic of this family of proteins.

Despite the similarity to the SKI/SNO/DAC domain, there are a number of important and significant differences. First, strand 2 of the SKI/SNO/DAC domain is not present in DNTTIP1, but is substituted by strand 4 from the carboxy-terminus of the domain. Second, there are additional helices at the amino- and carboxy-terminals of the domain in DNTTIP1 (Figure 3B).

### The SKI/SNO/DAC domain binds double-stranded DNA and nucleosomes

DACH1, a prototypical member of the SKI/SNO/DAC family, has been shown to bind double-stranded DNA and to function as a regulator of transcription by competing for DNA-binding with the transcriptional regulator Forkhead (40,42). DACH1 has a characteristically basic DNA-binding surface with a structural configuration reminiscent of the Winged-helix family of transcription factors with a short helix suitable for binding in the major groove of DNA. Other members of the SKI/SNO/DAC family such as SKI, do not have a basic DNA-binding surface and are not thought to bind to nucleic acids (41).

Inspection of the electrostatic surface of DNTTIP1 (calculated in APBS (45)) reveals that an equivalent region to DACH1 on DNTTIP1 has a strongly basic character suggestive of a similar role in DNA-binding (Figure 3D). DNTTIP1 also has an analogous helical segment (Helix 2) exposed on the surface of the protein, which could bind in the major groove of DNA. Helix 1 of DNTTIP1 also appears to contribute to the putative DNA binding surface and, by analogy with helix-turn-helix and homeodomain proteins, may interact with the phosphate backbone of the DNA.

**Figure 4.**
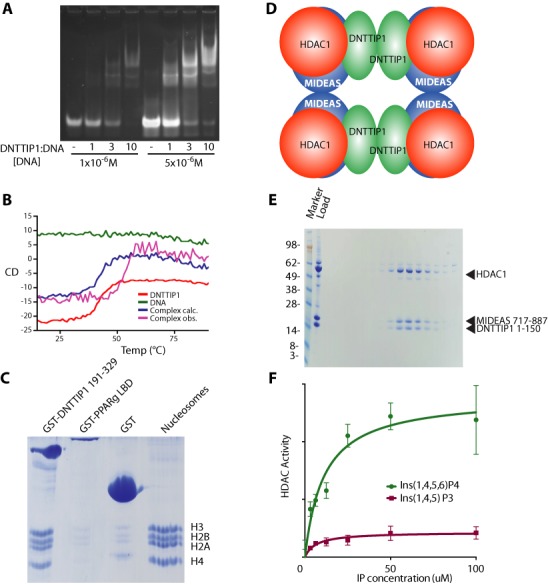
Function of the domains of DNTTIP1. (**A**) EMSA assay of DNTTIP1 (197–316) binding to a mixture of double stranded oligonucleotides. The DNA concentration and the ratio of DNTTIP1 to DNA are indicated. (**B**) Melting curves for DNTTIP1 (197–316), a mixture of double stranded oligonucleotides together with observed and calculated melting curves of DNTTIP1 bound to DNA. The circular dichroism was monitored at 222 nm. (**C**) SDS-PAGE stained with Coomassie of the binding of nucleosomes to GST-tagged DNTTIP1, GST-tagged PPARg LBD and just the GST-tag. (**D**) Schematic representation of the binding of DNTTIP1 (green) to the HDAC1 (orange) /MIDEAS (blue) complex. (**E**) Fractions from a Superdex-200 column of a complex of HDAC1, the ELM2-SANT domain of MIDEAS (717–887) and the N-terminus of DNTTIP1 containing the dimerization domain (1–150). (**F**) HDAC assay of the purified complex showing activation with inositol 1,4,5,6-tetraphosphate.

To test the hypothesis that the SKI/SNO/DAC domain in DNTTIP1 is a DNA-binding domain we performed a number of DNA-binding assays using mixed sequence DNA. Electrophoretic Mobility Shift Assays showed that DNTTIP1 binds to mixed sequence DNA with an affinity in the low micromolar range (Figure 4A). This affinity is comparable to the non-specific DNA-binding affinity of many transcriptional regulators. Thermal denaturation assays (monitored by CD spectroscopy) of the DNTTIP1 DNA-binding domain showed that DNA-binding increased the melting temperature of the SKI/SNO/DAC domain from 41 to 52°C (Figure 4B). This suggests that there is a significant structural stabilization on binding to DNA. Double-stranded DNA-binding activity was confirmed using NMR chemical-shift titrations with a ratio of DNA to protein of 0.25:1, 1:1 and 5:1 (Supplementary Figure S3). Significant broadening of the amide crosspeaks in an HSQC spectrum was observed when DNA was added at a ratio of 0.25:1 DNA:protein. This is likely to be the result of exchange broadening between bound and unbound species. Upon addition of an excess of DNA (5:1 DNA:protein) many crosspeaks reappeared, but with altered chemical shifts supporting the conclusion that there is a concerted conformational change upon DNA-binding.

To determine whether DNTTIP1 has a high affinity specific DNA-binding site, we performed several SELEX experiments with both full-length DNTTIP1 and the isolated SKI/SNO/DAC domain (43). However, after multiple rounds of selection, we were unable to enrich for a high affinity site. This suggests that the physiological role of the SKI/SNO/DAC domain in DNTTIP1 is to bind to DNA without specificity. This would fit well with the finding that DNTTIP1 is associated with a histone deacetylase complex (15,16) where it could act to mediate interaction with chromatin and other protein components could determine the specificity. Accordingly, we investigated if the DNTTIP1 SKI/SNO/DAC domain could bind to intact nucleosomes. Strikingly, when the SKI/SNO/DAC domain from DNTTIP1 was expressed with a GST-tag and immobilized on glutathione sepharose resin, it pulled-down near stoichiometric amounts of single nucleosomes, purified from avian blood (Figure 4C). This supports the hypothesis that DNTTIP1 is part of the chromatin binding module of the complex.

### Interaction of the dimerization domain of DNTTIP1 with the MIDEAS:HDAC1 complex

We recently solved the crystal structures of a SANT domain from SMRT bound to HDAC3 (10) and an ELM2-SANT domain from MTA1 in complex with HDAC1 (11). These structures established that there is a binding site for inositol phosphates at the interface between the SANT domain of the corepressors and the HDAC catalytic domains. The inositol phosphates are essential for maximal activity of the HDAC complex. Importantly, the HDAC1:MTA1 structure shows that the ELM2 domain of MTA1 not only interacts with HDAC1 but also mediates dimerization of the complex. To explore the oligomerization state of the HDAC1:MIDEAS:DNTTIP1 complex we purified a stable complex containing full-length HDAC1, the ELM2-SANT domain of MIDEAS (amino acids 717–887) and full-length DNTTIP1. We then used SEC-MALS to analyze the resulting complex (Supplementary Figure S4). The analysis showed that the complex exists as a single species with a molecular weight of 456 ± 14 kDa. This strongly suggests that the complex is a tetramer, with a calculated molecular weight of 448 kDa, containing four copies of each component. This is consistent with both DNTTIP1 and MIDEAS mediating dimerization such that the complex is a dimer of dimers (Figure 4D).

To determine whether it is the dimerization domain or chromatin binding domain of DNTTIP1 that mediates interaction with the HDAC1:MIDEAS complex, we attempted to purify a ternary complex containing either the dimerization domain (amino acids 1–150) or the carboxy-terminal chromatin binding domain (amino acids 150–329). We were only able to purify a complex with a construct containing the amino terminal dimerization domain (amino acids 1–150) (Figure 4E). When the carboxy terminal domain was co-expressed with HDAC1 and MIDEAS it was not possible to purify a complex suggesting that it is the amino-terminal domain that mediates interaction with the HDAC1:MIDEAS complex.

To confirm that the HDAC1:MIDEAS:DNTTIP1 complex is functionally active, we tested the activity of the complex in deacetylase assays. Since it has been shown that the HDAC3:SMRT and HDAC1:MTA1 complexes require inositol phosphates for their activity, we performed assays with increasing concentrations of inositol-1,4,5,6-tetrakisphosphate and inositol-1,4,5-triskisphosphate (10,11). We observed that the deacetylase activity of the MiDAC complex is enhanced by inositol-1,4,5,6-tetrakisphosphate, but not inositol-1,4,5-triskisphosphate which is in line with previous findings with the other HDAC complexes (Figure 4F) (11).

**Figure 5.**
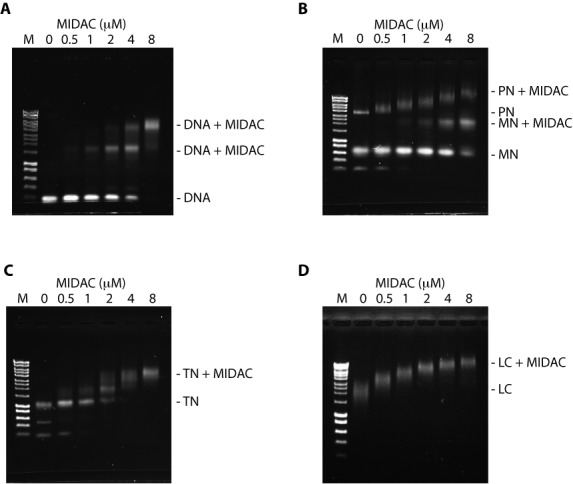
Chromatin binding of the complex with full-length HDAC1, full-length DNTTIP1 and MIDEAS (650–887). Electrophoretic Mobility Shift Assays of complex at the indicated concentrations binding to: (**A**) 0.38 μM 147 base pairs double stranded DNA; (**B**) 0.62 μM 167 base pairs of 601 sequence reconstituted with histone octamer (MN – mononucleosome) using pUC DNA as competitor (PN – polynucleosome)); (**C**) 0.18 μM 501 base pairs (167 × 3) of 601 sequence reconstituted with histone octamer (TN – tri nucleosome) using 147 bp DNA as competitor; (**D**) 0.4 A260/ml of long chromatin extracted from HEK293F cells and purified using a 5–50% sucrose gradient. DNA markers (1 kb Hyperladder Bioline) are marked with M.

To explore the ability of the entire MiDAC complex to bind chromatin, we purified full-length HDAC1, with full-length DNTTIP1 and MIDEAS (amino acids 650–887) (Supplementary Figure S5). Electrophoretic Mobility Shift Assays using naked DNA as well as mono-, tri- and poly-nucleosomes revealed that the complex is able to bind to naked DNA and the three chromatin species tested (Figure 5A–C). The complex seems to bind most avidly to poly-nucleosomes assembled on pUC DNA (Figure 5B) and we therefore tested the ability of the complex to bind to long chromatin purified from HEK293F nuclei (Figure 5D). All the long chromatin was shifted at a MiDAC concentration of 0.5 μM supporting the finding that the complex preferentially binds to long nucleosome arrays, probably due to the availability of multiple binding sites. It remains to be seen whether the complex is just binding to the nucleic acid or whether there are interactions with histones.

## DISCUSSION

Three independent proteomics studies recently showed that, in addition to the well-studied NuRD, CoREST and Sin3A complexes, HDAC1 also forms the catalytic subunit of a novel cyclin A associated complex that involves the proteins DNTTIP1 and MIDEAS (15–17). We have determined the structure of two domains from DNTTIP1. The amino terminal domain is a dimerization domain that mediates interaction with the HDAC1:MIDEAS complex. The carboxy terminal domain is structurally similar to the SKI/SNO/DAC domain and mediates interaction of the complex with chromatin.

Multi-angle laser light scattering data show that the intact MiDAC complex forms a tetramer with four copies of each of the component proteins. This can be explained through the HDAC1:MIDEAS complex forming a dimer analogous to the HDAC1:MTA1 complex and that the dimerization domain of DNTTIP1 mediates dimerization of the dimeric complex forming the full tetramer. The role of the DNTTIP1 therefore appears to be not only to target chromatin but also to act as a scaffold protein for the complex. Oligomerization of transcriptional repression complexes is emerging as a common theme. The HDAC1:MTA1 complex is a dimer mediated by MTA1; the SMRT/NCoR complex is assembled around the tetrameric scaffold protein TBL1 and TLE1 mediates tetramerization of the Groucho repression complex (9,44). The finding that scaffold proteins assemble multiple HDAC catalytic units into multi-valent complexes fits well with the requirement to deacetylate multiple histone tails in order to regulate gene expression through concerted changes in chromatin structure.

Another common feature of HDAC complexes is that there are often multiple versions of the co-repressor protein that directly interacts with the HDAC catalytic unit. There are three MTA proteins 1–3 and three CoREST proteins 1–3. There are also three versions of the MIDEAS-like proteins called MIDEAS, ZNF541 and TRERF1. The homology between these proteins is restricted to the highly conserved ELM2-SANT domain and a moderately conserved region of 80 amino acids amino-terminal to the ELM2-SANT domain. Outside this conserved region, the proteins show very little sequence homology and given their different patterns of expression, presumably have tissue specific functions that may be associated with different targeting of these complexes in the cell. ZNF541 is a protein that is associated with spermatogenesis and is found upregulated in testis (17). MIDEAS is upregulated in cells blocked in mitosis by nocodazole and is found associated with Cyclin A in the cell cycle (15,18). In mice, transcripts of TRERF1 were found to be highest in the brain, thymus and testis (24).

The finding that the SKI/SNO/DAC domain from DNTTIP1 mediates interaction with both naked DNA and nucleosomes suggests that it serves as a chromatin interaction or substrate recruitment domain for the MiDAC complex. We show that the intact tetrameric MiDAC complex is able to interact with naked DNA, mono- and tri-nucleosomes, polynucleosomes bound to linearized pUC plasmid and also long chromatin isolated from HEK293F nuclei. Since the MiDAC complex binds to naked DNA, it is possible that MiDAC complex might be binding to the linker DNA in the various chromatin contexts. However, it appears that the MiDAC complex binds most avidly to the longer chromatin suggesting that this is a closer approximation to the natural target of this complex.

In conclusion, we have shown that DNTTIP1 is a dimeric protein required for the stable assembly of the cyclin A associated MiDAC complex. The three proteins HDAC1, MIDEAS and DNTTIP1 form a large complex assembly containing four copies of each protein. In addition to contributing to the assembly of this complex, DNTTIP1 also mediates a chromatin-binding activity that is likely to be important for substrate presentation to the active catalytic site of HDAC1.

## ACCESSION NUMBERS

The coordinates and diffraction data of the dimerization domain have been deposited with the Protein Data Bank with the ID 4d6k. The DNA binding domain NMR structure and NMR constraints have been deposited with the relevant databases with the IDs: PDB 2mwi and BMRB 25326.

## SUPPLEMENTARY DATA

Supplementary Data are available at NAR online.

SUPPLEMENTARY DATA
