# Supplementary material for: Structural and functional characterization of a cell cycle associated HDAC1/2 complex reveals the structural basis for complex assembly and nucleosome targeting
Source: Nucleic Acids Res. 2015 Feb 4;43(4):2033–44. doi: 10.1093/nar/gkv068 (PMC4344507; doi:10.1093/nar/gkv068)
Supplement: SUPPLEMENTARY DATA [file supp_43_4_2033__index.html]

Structural and functional characterization of a cell cycle associated HDAC1/2 complex reveals the structural basis for complex assembly and nucleosome targeting — Structural and functional characterization of a cell cycle associated HDAC1/2 complex reveals the structural basis for complex assembly and nucleosome targeting — SUPPLEMENTARY DATA 

# Structural and functional characterization of a cell cycle associated HDAC1/2 complex reveals the structural basis for complex assembly and nucleosome targeting

## SUPPLEMENTARY DATA

**Files in this Data Supplement:**

- SUPPLEMENTARY DATA
