# Supplementary material for: Structural and functional characterization of a cell cycle associated HDAC1/2 complex reveals the structural basis for complex assembly and nucleosome targeting
Source: Nucleic Acids Res. 2015 Feb 4;43(4):2033–44. doi: 10.1093/nar/gkv068 (PMC4344507; doi:10.1093/nar/gkv068)
Supplement: SUPPLEMENTARY DATA [file supp_gkv068_nar-02844-m-2014-File008.pdf]

Supplementary Table 1. Data collection, phasing and refinement statistics for MAD (SeMet) structures

	Native		SeMet	
**Data collection**				
Space group	$P2_1 2_1 2_1$		$P2_1 2_1 2_1$	
Cell dimensions				
a, *b*, *c* (Å)	54.91, 103.05, 108.93		55.12, 103.12, 110.99	
α, β, γ (°)	90.0, 90.0, 90.0		90.0, 90.0, 90.0	
		Peak	*Inflection*	*Remote*
Wavelength		0.9805	0.9808	0.9537
Resolution (Å)	103.14-2.10 (2.21-2.10)	110.43-2.75 (2.90-2.75)	110.99-2.75 (2.90-2.75)	110.43-2.75 (2.90-2.75)
R_{merge}	8.5 (29.1)	9.7 (30.3)	9.3 (32.8)	9.3 (34.8)
$I / \sigma I$	11.9 (3.4)	8.8 (4.0)	9.2 (4.1)	9.2 (3.9)
Completeness (%)	98.9 (95.3)	99.6 (99.8)	99.7 (99.8)	99.7 (99.8)
Redundancy	3.6 (3.3)	3.3 (3.4)	3.3 (3.4)	3.3 (3.4)
**Refinement**				
Resolution (Å)	2.10			
No. reflections	34537			
R_{work} / R_{free}	19.5/22.4			
No. atoms	3504			
Protein	3375			
Water	129			
B-factors				
Protein	36.4			
Water	40.1			
R.m.s deviations				
Bond lengths (Å)	0.016			
Bond angles (°)	1.699			

*Values in parentheses are for highest-resolution shell.

Supplementary Table 2. NMR constraints and structural statistics

Total NOE distance constraints	2781
Short Range (i, i-1)	1283
Medium Range (i, i- < 5)	651
Long Range (i, i- ≥ 5)	847
Total dihedral angle constraints	166
ψ	83
Φ	83
Average Cyana target function	2.18 ± 0.34
Violations	
Maximum distance violation (Å)	0.49
Maximum van der Waals violation (Å)	0.28
Maximum dihedral angle violation (°)	4.97
RMS deviations from mean structure*	
Backbone atoms (N, Cα, C') (Å)	0.86 ± 0.2
All heavy atoms (Å)	1.29 ± 0.19
Ramachandran plot	
Residues in most favourable regions (%)	75.9
Residues in additionally allowed regions (%)	21.8
Residues in generously allowed regions (%)	1.9
Residues in disallowed regions (%)	0.5**

*residues 199-315 (residues that showed medium range or longer NOEs)
** These residues, apart from M258, had no chemical shift assignments.

Supplementary Table 3. Mass spectrometry data for FLAG tagged DNTTIP1

Protein ID	Peptide sequence	Mascot Ion score
TDIF1_HUMAN	AVLQPSINEEIQTVFNK	114
TDIF1_HUMAN	DLAASDDYR	28.8
TDIF1_HUMAN	DNVGEEVDAEQLIQEACR	97.7
TDIF1_HUMAN	GGLELGDAGAAGQLVLTNPWNIMIK	66.2
TDIF1_HUMAN	GGLELGDAGAAGQLVLTNPWNIMIK	93.8
TDIF1_HUMAN	GRPPGHILSSDR	28.8
TDIF1_HUMAN	HWLAEQHHMR	31.9
TDIF1_HUMAN	KGRPPGHILSSDR	37
TDIF1_HUMAN	KYMETLR	36.2
TDIF1_HUMAN	LNESTTFVLGSR	96.2
TDIF1_HUMAN	MAYLLIEEDIRDLAASDDYR	49
TDIF1_HUMAN	SQMTTSFTDPAISMDLLR	103
TDIF1_HUMAN	SQMTTSFTDPAISMDLLR	123
TDIF1_HUMAN	YAADPQDKHWLAEQHHMR	61.3
CN043_HUMAN	IPGTDAQAQAEDMNVK	43.1
CN043_HUMAN	KEGEEEVPEIQEKEEQEEGR	24.9
CN043_HUMAN	SAQEEVEVDIK	43
CN043_HUMAN	SHESNAPGSAGGQASEKPR	42.1
CN043_HUMAN	WPNSVMAPGR	33.5
HDAC1_HUMAN	SFNLPMLMLGGGGYTIR	62.0
HDAC1_HUMAN	VKTEDEKEKDPEEK	18.6

Modified amino-acids shown in red

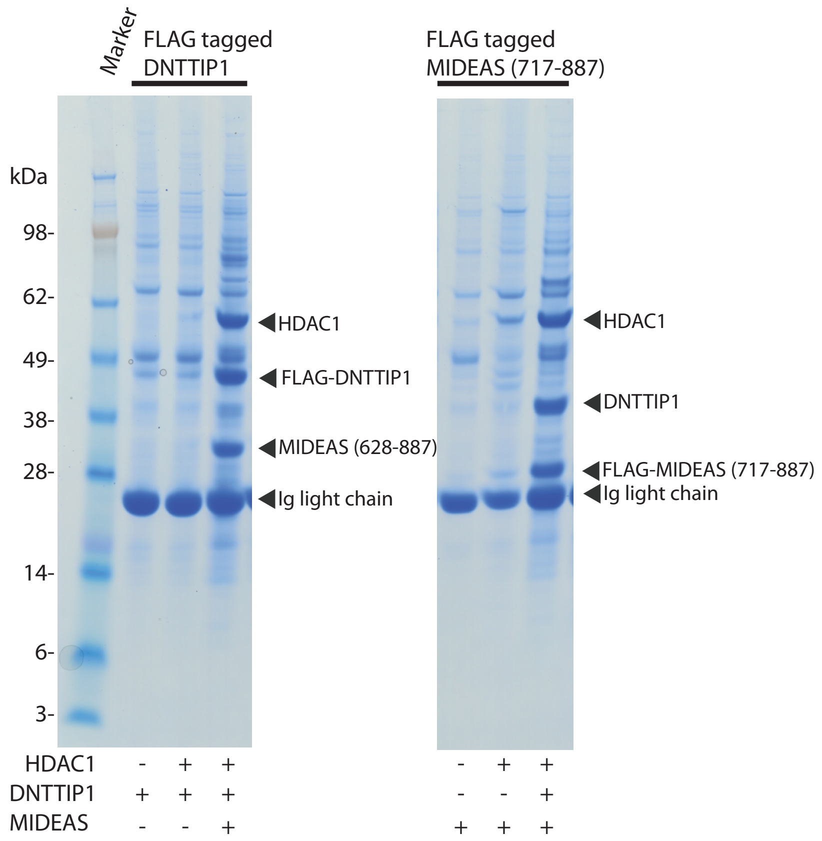

Supplementary Figure 1

A stable complex of HDAC1, DNTTIP1 and MIDEAS.

Small scale co-transfection of HEK293F cells and purification of FLAG tagged constructs of MIDEAS and DNTTIP1 with DNTTIP1, MIDEAS and HDAC1 as indicated.

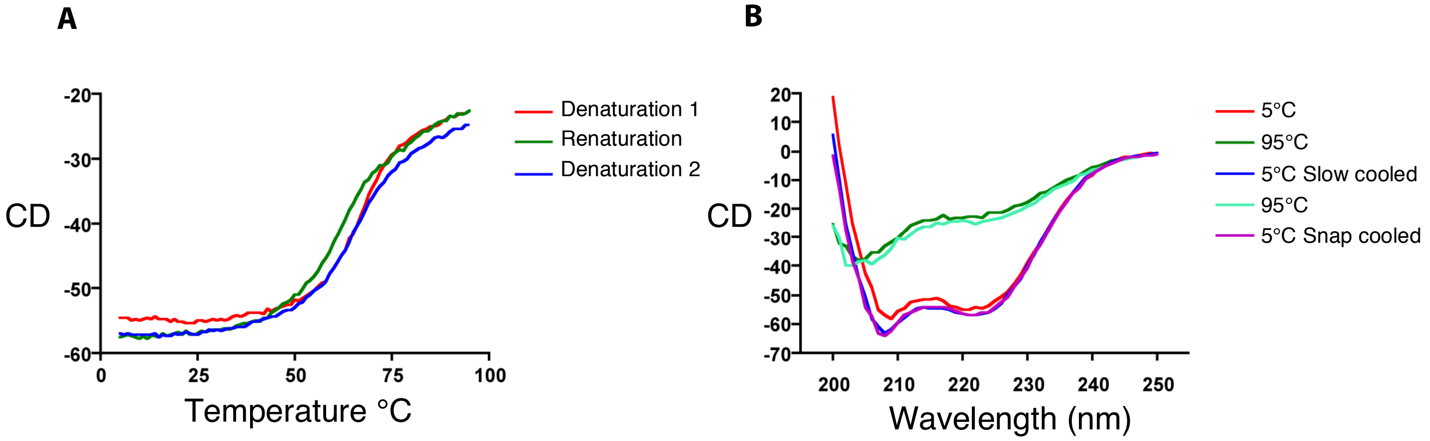

Supplementary Figure 2

Circular dichroism experiments on the dimerisation domain of DNTTIP1.

(A) Melting curves of DNTTIP1 (56-147) as it is thermally denatured, slow cooled and then denatured again. The circular dichroism was monitored at 222 nm. (B) Circular dichroism spectra of DNTTIP1 (56-147) at 5°C before, after slow cooling and after snap cooling together with spectra at 95°C after both rounds of thermal denaturation.

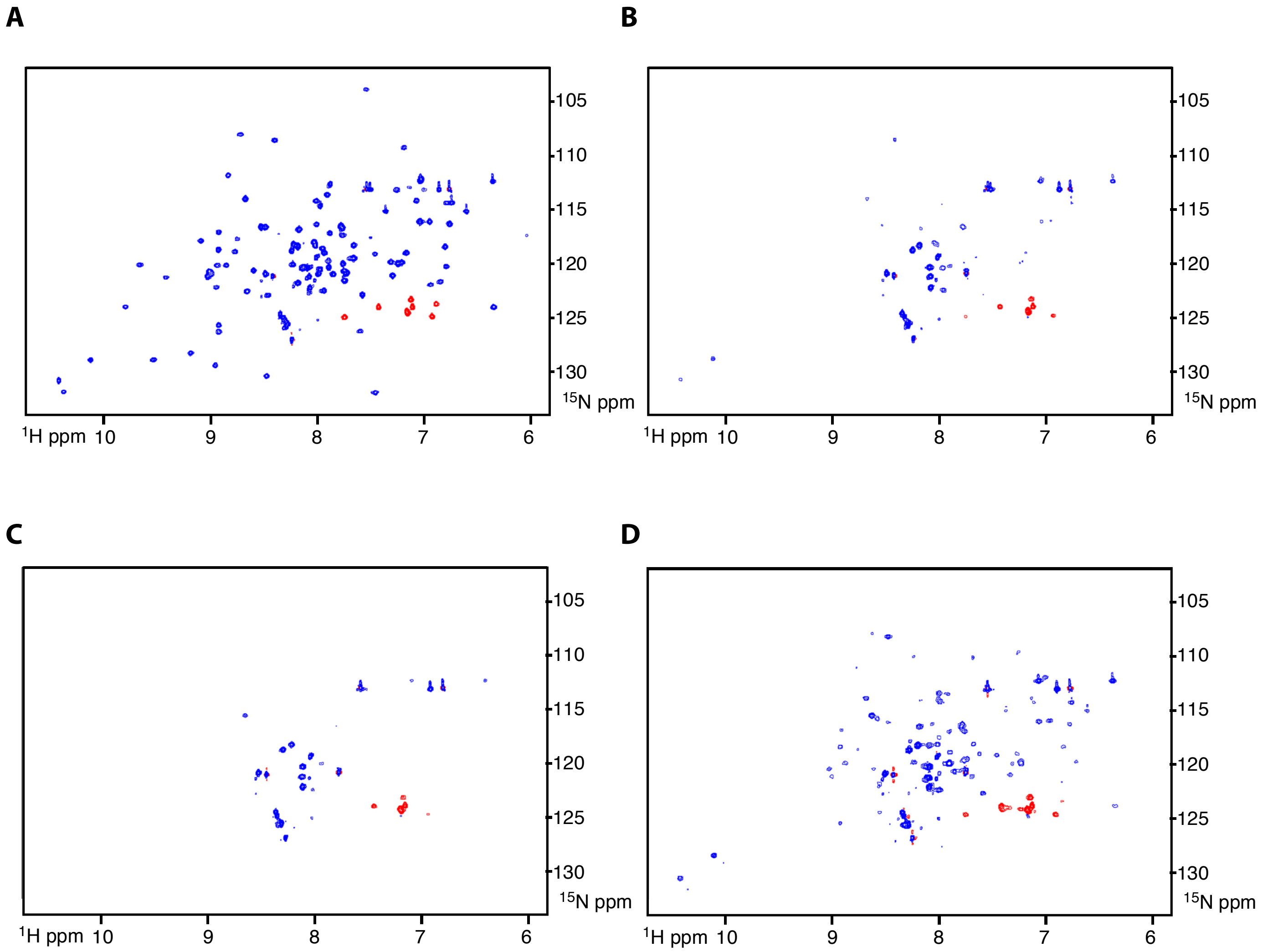

Supplementary Figure 3

15N-HSQC spectra of the DNA titration with DNTTIP1.

A) 0.2mM DNTTIP1 with no DNA, B) with 0.25 molar equivalents of DNA, C) with 1.0 molar equivalents of DNA, D) with 5 molar equivalents of DNA. Red peaks are arginine sidechain NE resonances folded from ~ 70ppm.

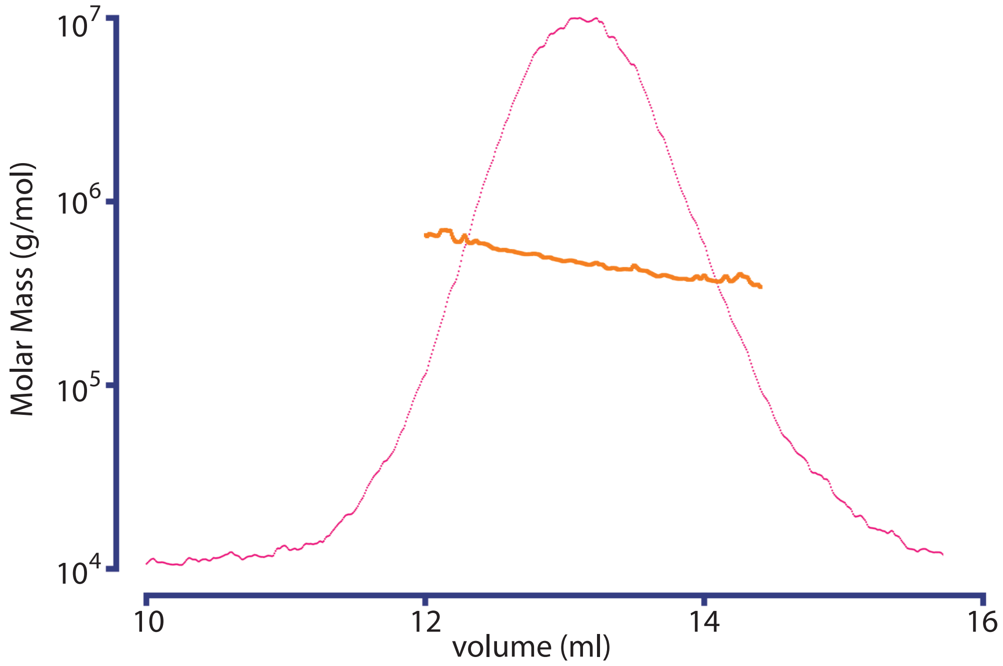

Supplementary Figure 4
SEC-MALS profile of the complex with full-length HDAC1, full-length DNTTIP1 and MIDEAS (717-887).
The measured molecular weight is 455.9 kDa +/-14 kDa. The calculated molecular weight for a tetramer containing four copies of each protein is 448 kDa.

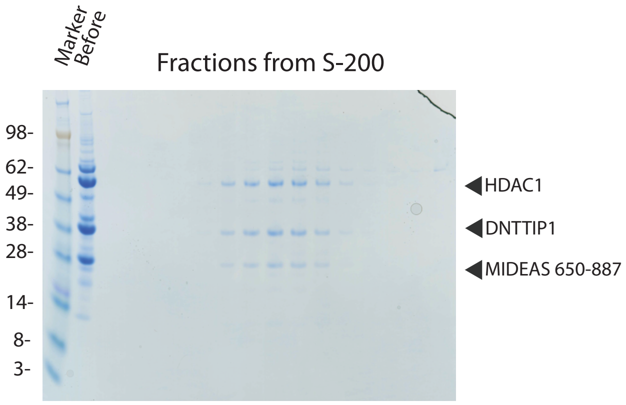

Supplementary Figure 5
Superdex S-200 gel filtration purification of the full length HDAC1, full length DNTTIP1 and MIDEAS(650-887) complex.
